# Interferon gamma polymorphisms and hepatitis B virus-related liver cirrhosis risk in a Chinese population

**DOI:** 10.1186/s12935-015-0184-2

**Published:** 2015-03-31

**Authors:** Yifan Sun, Yu Lu, Li Xie, Yan Deng, Shan Li, Xue Qin

**Affiliations:** Department of Clinical Laboratory, First Affiliated Hospital of Guangxi Medical University, 6 Shuangyong Road, Nanning, 530021 Guangxi People’s Republic of China

**Keywords:** Interferon gamma, Polymorphisms, Hepatitis B virus, Hepatitis B virus

## Abstract

**Background:**

Previous studies proved that interferon gamma (IFN-γ) gene polymorphisms were associated with the risk of hepatitis B virus (HBV) infection. However, the association between *IFN-γ* polymorphisms and HBV-related liver cirrhosis (HBV-LC) risk is still unclear.

**Methods:**

*IFN-γ* +874 T/A and +2109G/A genotypes were determined in 126 HBV-LC patients, 129 chronic hepatitis B(CHB) patients, and 173 early HBV infection controls using a sequence-specific primer-polymerase chain reaction and a polymerase chain reaction-restriction fragment length polymorphism, respectively.

**Results:**

Significant associations were observed between +2109A/G polymorphisms and HBV-LC risk in the co-dominant model (GG vs. AA: OR = 0.321, 95% CI = 0.130-0.793, *P* = 0.014), the allelic model (OR = 0.565, 95% CI = 0.388-0.825, *P* = 0.003), the dominant model (OR = 0.551, 95% CI = 0.344-0.883, *P* = 0.013), and the recessive model (OR = 0.385, 95% CI = 0.159-0.930, *P* = 0.034). In addition, haplotype analysis indicated that the T^+874^G^+2109^ haplotype significantly decreased the HBV-LC risk (OR = 0.106, 95% CI = 0.022-0.502, *P* = 0.000), and A^+874^A^+2109^ haplotype significantly increased the LC risk (OR = 1.485, 95% CI = 1.065-2.070, *P* = 0.019). No significant associations were observed between *IFN-γ* +874 T/A polymorphisms and HBV-LC risk, as well as the two single-nucleotide polymorphisms (SNPs) and CHB risk (*P* > 0.05).

**Conclusions:**

Our observations suggested a significant association of *IFN-γ* polymorphisms with HBV-LC risk in the Chinese population.

## Introduction

Hepatitis B virus (HBV) infection is prevalent in the Chinese population and is associated with a variety of clinical consequences. Some patients become asymptomatic carriers while others develop liver cirrhosis (LC) during the chronic phase, which finally develops into hepatocellular carcinoma [1]. Persistent infection with HBV is strongly associated with the development of LC. However, only a minority of lifelong chronic carriers of HBV will eventually develop LC, and the molecular and cellular mechanisms of LC pathogenesis are still not completely understood [2]. In the context of chronic liver disease, gene polymorphisms, such as the single-nucleotide polymorphism (SNP), have been considered a risk factor for LC [3].

Cytokines play a fundamental role in the immunopathogenesis of HBV related diseases. Our previous studies found that cytokines gene polymorphisms, such as interleukin 4 (IL4), IL16, IL27, and IL23, were associated with the risk of HBV infection and HBV-related HCC [4-7]. Interferon gamma (IFN-γ) is a T-helper 1(Th1) pro-inflammatory cytokine that plays a pivotal role in antiviral activities and has antitumor and antiproliferative effects [8]. The *IFN-γ* gene on chromosome 12q24 spans approximately 5.4 kb and is composed of four exons, with three introns [8]. The SNPs in the *IFN-γ* gene region can influence the IFN-γ production, which may increase the risk of viral infection [9].The IFN-γ +874 T/A (rs2430561) in the first intron of the *IFN-γ* gene, in which the TT genotype produces a high level of IFN-γ, helps the host’s defense against viral infection. Conversely, the genotypes AA and AT cause low IFN-γ production, which may increase the risk of viral infection. Moreover, the DNA sequences containing the +874 T/A and +2109A/G (rs1861494) are the specific binding sites for the NF-κB transcription factor. If the pathway is affected, it may lead to oxidative damage, which may also increase the risk of cirrhosis [9].

As previously reported, genetic variations in IFN-γ may be related to virus-related diseases, such as cervical cancer caused by human papillomavirus infection [10] and leprosy caused by Mycobacterium leprae [11]. The IFN-γ gene polymorphisms are also widely studied in HBV infection and HBV-HCC [12-14]. Recent studies observed the association between the IFN-γ polymorphisms and Hepatitis C virus related LC risk [15,16]. In this report, we hypothesis that IFN-γ gene polymorphisms are considered to be strongly related to the development of HBV-LC, and design a case–control study to examine the association of the IFN-γ +874 T/A and +2109A/G polymorphisms with HBV-LC risk in a Chinese population.

## Materials and methods

### Study subjects

In total, 173 early HBV infection controls, 126 HBV-LC patients, and 129 chronic hepatitis (CHB) patients were enrolled in this study. All participants were recruited at the First Affiliated Hospital of Guangxi Medical University between May 2013 and April 2014. The inclusion criteria for the HBV-LC group were as follows: (1) the hepatitis B surface antigen and the hepatitis B virus core antibody were positive; (2) the pathological examination was confirmed; and (3) no other hepatitis virus infections, such as hepatitis C (HCV) or hepatitis E (HEV), were present. The early HBV infection control group that tested only hepatitis B surface antigen positive without any other diseases was carefully matched to the HBV-LC group for gender, smoking and alcohol consumption. CHB was further diagnosed with serum HBV-DNA levels >1,000 copies/mL and elevated alanine aminotransferase or aspartate aminotransferase (>2 times the upper limit of normal).

Written informed consent was obtained from each participant for the use of their DNA. The study was approved by the official recommendations of the ethics committee of the First Affiliated Hospital of Guangxi.

### DNA extraction and genotyping

Peripheral blood was collected in ethylenediaminetetraacetic acid tubes. Genomic DNA was extracted from whole blood using a QIAamp DNA blood mini kit (QIAGEN GmbH, Hilden, Germany) and stored at −20°C.

The *IFN-γ* +874 T/A polymorphism was determined by a sequence-specific primer-polymerase chain reaction according to Dai et al. [15]. The polymorphism of *IFN-γ* +2109A/G was determined by a polymerase chain reaction-restriction fragment length polymorphism-based method according to Sarvari et al. [16], with some modifications. Briefly, polymerase chain reactions (PCR) were conducted with 2.0 uL of genomic DNA, 1.0 uL of each specific primer, and 10 uL of 2x of Dream Taq Green PCR Master Mix (Fermentas, Burlington, Canada). Genotyping of the SNP at +874 included a second primer pair (0.15 lM each) used as an internal control for amplification. A 303-bp T or A allele-specific product and a 400 bp internal control product were present when successful PCR amplification occurred. The following digested fragments were obtained for *IFN-γ* +2109A/G with *MspI* restriction endonuclease: AA, 267 bp; AG, 267 + 245 + 22 bp; GG, 245 + 22 bp. The primer sequences and reaction condition are shown in Table 1. PCR reactions were repeated at least once when the results were unclear.

**Table 1:** **The primer sequences and reaction conditions**

**Locus**	**Primer**	**Annealing temperature**	**Method**	**Product size(bp)**
+874 T/A	T allele primer: 5′-TTCTTACAACACAAATACAATACT-3′	61°C,56°C	SSP-PCR	AA,AT,TT:303
(rs2430561)	A allele primer: 5′-TTCTTACAACACAAATACAATACA-3′			
	common primer: 5′-TCCCATATAAACCTATAATATGCA-3′			
	inner reference F: 5′-CTTCAACACCCCAGCCTAGTAC-3′			Inner reference: 400
	inner reference R: 5′-CCTCAGGGCAGCGAGACC-3′			
+2109A/G (rs1861494)	F: 5′-GAGAGGTAAGGTGAGAGAGAACC-3′	55°C	RFLP-PCR (MspI)	AA:267
	R: 5′-AGCAAAGAAGGTCTACAAACT-3′			AG:267 + 245 + 22
				GG:245 + 22

SSP-PCR,sequence-specific primer-polymerase chain reaction; RFLP-PCR, polymerase chain reaction-restriction fragment length polymorphism.

Afterwards, about 10% of the samples remained to be confirmed by DNA sequencing with an ABI Prism 3100 (Shanghai Sangon Biotech Co., Ltd., China), and a 100% concordance rate was achieved.

### Statistical analysis

In this study, the differences in genotype distributions, the allele frequencies, and the Hardy–Weinberg equation (HWE) were tested by chi-square analysis and Fisher’s exact test as appropriate. The differences in age were tested by Student’*t*-test. An association between the genotypes and HBV-LC risk was calculated as odds ratios (ORs), with 95% confidence intervals (CIs), after logistic regressive analysis by adjusting for confounding factors (age, gender, alcohol consumption, etc.). Haplotype analysis was performed using PLINK software. All of the data were calculated using SPSS 19.0 software (IBM Corporation, Armonk, NY, USA). All statistical tests were two-tailed, and *P* <0.05 was considered statistically significant.

## Results

### Characteristics of the study subjects

No significant differences in gender distribution, smoking and alcohol consumption were identified between cirrhotic patients and the control group. However, the HBV-LC patients were, on average, 10 years older than healthy controls and CHB patients, with the difference being statistically significant(*P* = 0.000). Characteristics of the HBV-LC patients and control subjects included in this study are shown in Table 2.

**Table 2:** **The basic characters of the study population**

**Factors**	**Control**	**CHB**	**LC**	***P*** **value**
	**N = 173**	**N = 129**	**N = 126**	
Age(mean ± SD)*	37.78 ± 11.71	37.71 ± 11.55	46.55 ± 10.28	0.000
Gender**				
Male	149 (86.1%)	115 (89.1%)	106 (86.4)	0.497
Femle	24 (13.9%)	14 (10.9%)	20 (13.6)	
Smoking**				
No	127 (73.4%)	96 (74.4)	101 (80.2)	0.373
Yes	46 (26.6%)	33 (25.6)	25 (19.8)	
Alcohol**				
No	121 (69.9%)	88 (68.2)	91 (70.1)	0.782
Yes	52 (30.1%)	41 (31.8)	35 (29.9)	

*Student’ *t* test.

**Chi-square test.

### Genotype and allele frequency distributions of the IFN-γ polymorphisms

Genotype and allele frequencies of the *IFN-γ* +874 T/A and +2109A/G polymorphisms between the case and the control subjects are shown in Table 3. The two SNP polymorphisms were consistent with the HWE (*P* > 0.05). Statistical analysis of the allele frequencies demonstrated that the +2109A/G G allele was lower in the case subjects than in the controls (*χ*^2^ = 8.681; *P* = 0.013). However, no other genotype and allele frequency distributions were observed as discriminatory between the case and the control subjects (*P* > 0.05).

**Table 3:** **Frequency distributions of** ***INF-γ*** **polymorphisms among cases and controls**

**Polymorphisms**		**Frequencyn (%)**			**CHB vs. Control**		**HBV-LC vs. Control**	
		**LC = 126**	**CHB = 129**	**Control = 173**	***P***	**OR (95% CI)**	***P***	**OR (95% CI)**
**+874(T/A)**								
Genotypes								
	AA	90 (71.4)	84 (65.1)	116 (67.1)		1^Ref^		1^Ref^
	AT	32 (25.4)	36 (27.9)	47 (27.2)	0.781	1.086 (0.607-1.943)	0.588	0.863 (0.507-1.470)
	TT	4 (3.2)	9 (7.0)	10 (5.8)	0.481	1.458 (0.511-4.159)	0.264	0.505 (0.152-1.675)
Allele								
	A	212 (84.1)	204 (79.1)	279 (80.6)		1^Ref^		1^Ref^
	T	40 (15.9)	54 (20.9)	67 (19.4)	0.480	1.176 (0.750-1.844)	0.250	0.775 (0.505-1.196)
Dominant model AT + TT vs AA					0.617	1.148 (0.668-1.974)	0.389	0.801 (0.483-1.328)
Recessive model TT vs AA + AT					0.505	1.421 (0.506-3.991)	0.291	0.526 (0.160-1.730)
**+2109 (A/G)**								
Genotypes								
	AA	79 (62.7)	72 (55.8)	82 (47.4)		1^Ref^		1^Ref^
	AG	40 (32.7)	43 (33.3)	68 (39.3)	0.640	0.874 (0.498-1.534)	0.071	0.630 (0.381-1.041)
	GG	7 (5.6)	14 (10.9)	23 (13.3)	0.256	0.625 (0.278-1.407)	0.014	0.321 (0.130-0.793)
Allele								
	A	198 (78.6)	187 (72.5)	232 (67.6)		1^Ref^		1^Ref^
	G	54 (21.4)	71 (27.5)	111 (32.4)	0.232	0.785 (0.528-1.167)	0.003	0.565 (0.388-0.825)
Dominant model AG + GG vs AA					0.392	0.799 (0.478-1.336)	0.013	0.551 (0.344-0.883)
Recessive model GG vs AA + AG					0.295	0.659 (0.301-1.440)	0.034	0.385 (0.159-0.930)

LC, liver cirrhosis; CHB, chronic hepatitis; Ref, reference; OR, odds ratio; 95% CI, 95% confidence interval.

*P* value: after logistic regressive analysis by adjusting for confounding factors (age, gender, alcohol consumption, etc.).

### Association between the IFN-γ polymorphisms and HBV-related diseases

The association between the *IFN-γ* polymorphisms and HBV-related diseases is shown in Table 3. In the logistic regression analyses after adjusting for age, gender, and smoking and drinking status, the *IFN-γ* +874 T/A and +2109A/G polymorphisms were not associated with CHB risk under all comparison models (*P* > 0.05). Significant associations were observed between +2109A/G polymorphisms and HBV-LC risk in the co-dominant model (GG vs. AA: OR = 0.321, 95% CI = 0.130-0.793, *P* = 0.014), the allelic model (G allele vs. A allele: OR = 0.565, 95% CI = 0.388-0.825, *P* = 0.003), the dominant model (GG + AG vs. AA: OR = 0.551, 95% CI = 0.344-0.883, *P* = 0.013), and the recessive model (GG vs. AG + AAOR = 0.385, 95% CI = 0.159-0.930, *P* = 0.034).

### Haplotype analyses

As the haplotype-based analysis is considered to have greater power than SNP genotyping, linkage disequilibrium (LD) and haplotype analysis for all patient groups and healthy controls was further conducted using PLINK software. A minor LD was found between the two studied SNPs (D’ = 0.038, *r*^2^ = 0.000).The haplotype distribution results are shown in Table 4. Four haplotypes were derived from the observed genotypes. According to the results, the A^+874^A^+2109^ haplotype was the most common haplotype, representing >50% in all groups, followed by A^+874^G^+2109^ and then T^+874^G^+2109^. T^+874^G^+2109^ was a protective haplotype that significantly decreased HBV-LC risk (OR = 0.106, 95% CI = 0.022-0.502, *P* = 0.000) while A^+874^A^+2109^ haplotype significantly increased the HBV-LC risk (OR = 1.485, 95% CI = 1.065-2.070, *P* = 0.019).

**Table 4:** **Haplotype analysis among +874 T/A and +2109A/G SNPs**

**Haplotype**	**CHB vs control**				**LC vs control**			
	**Case (%)**	**Control (%)**	***P***	**OR [95% CI]**	**Case (%)**	**Control (%)**	***P***	**OR [95% CI]**
A^+874^A^+2109^	148 (57.4)	186 (53.8)	0.384	1.155 [0.835 ~ 1.599]	160 (63.4)	186 (53.8)	0.019	1.485 [1.065 ~ 2.070]
A^+874^G^+2109^	56 (21.7)	93 (26.8)	0.148	0.756 [0.517 ~ 1.105]	52 (20.7)	92 (26.8)	0.087	0.714 [0.485 ~ 1.051]
T^+874^A^+2109^	39 (15.1)	46 (13.2)	0.512	1.166 [0.735 ~ 1.850]	38 (15.2)	45 (13.2)	0.495	1.175 [0.739 ~ 1.868]
T^+874^G^+2109^	15 (5.8)	21 (6.1)	0.876	0.948 [0.480 ~ 1.872]	2 (0.7)	21 (6.1)	0.001	0.106 [0.022 ~ 0.502]

### Genotype and allele frequencies among different races

Considering that the genetic background could be distinct among different populations, we compared the genotype and allele frequencies of the two SNPs in our control group with those in different races according to the Haplotype Map (HapMap) project (http://www.ncbi.nlm.nih.gov/snp/). However, no data from different races were obtained from HapMap for rs2430561. Thus, we compared the genotype and allele frequencies of the +874 T/A SNP according to previous studies [14,17-19]. The data are shown in Table 5. The results indicated that significant differences were found in the genotype frequencies of the +874 T/A SNP in our study unlike in the studies from Korea [19], Brazil [17], India [14], and Tunisia [18] (all P < 0.05). Furthermore, the T allele frequencies of the +874 T/A SNP were similar to those observed in the Korean population [19] (P = 0.553) but were inconsistent with those in various other ethnic populations [14,17,18] (all P < 0.05). For the +2109A/G SNP, our results showed similar genotype and allele frequencies to those in HCB (Han Chinese in Beijing, China) and CEU (Utah residents with Northern and Western European ancestry) but were significantly different from those in JPT (Japanese in Tokyo, Japan) and YRI (Yoruba in Ibadan, Nigeria) (all P < 0.05).

**Table 5:** **Genotype frequencies in healthy control subjects in this study and from the HapMap project or previous studies**

**SNPs**	**Number**	**Genotype frequency**			***P***	**Allele frequency**		***P***
**+874 T/A**		**AA**	**AT**	**TT**		**A**	**T**	
This study	173	116 (67.1)	47 (27.2)	10 (5.8)	1^Ref^	279 (80.6)	67 (19.4)	1^Ref^
Korea [20]	201	151 (75.1)	47 (23.4)	3 (1.5)	0.043	249 (82.5)	53 (17.5)	0.553
Brazil [18]	202	79 (39.1)	82 (40.6)	41 (20.3)	0.000	240 (59.4)	164 (40.6)	0.000
India [15]	146	52 (35.6)	77 (52.7)	17 (11.6)	0.000	181 (62.0)	111 (38.0)	0.032
Tunisia [19]	103	33 (32.0)	47 (45.6)	23 (22.3)	0.000	113 (54.9)	93 (45.1)	0.000
**+2109A/G**		**AA**	**AG**	**GG**		**A**	**G**	
This study	173	112 (64.7)	52 (30.1)	9 (5.2)	1^Ref^	232 (67.6)	111 (32.4)	1^Ref^
HCB	45	21 (46.7)	18 (40.0)	6 (13.3)	0.040	60 (66.7)	30 (33.3)	0.008
JPT	44	16 (36.4)	13 (29.5)	15 (34.1)	0.000	45 (51.1)	43 (48.9)	0.000
CEU	58	23 (39.7)	31 (53.4)	4 (6.9)	0.003	77 (66.4)	39 (33.6)	0.003
YRI	59	47 (79.7)	12 (20.3)	0 (0)	0.050	106 (89.8)	12 (10.2)	0.013

*HCB* Han Chinese in Beijing, China, *JPT* Japanese in Tokyo, Japan, *CEU* Utah residents with Northern and Western.

European ancestry, *YRI* Yoruba in Ibadan, Nigeria.

## Discussion

Cytokine SNPs causing modulated expression of the encoded protein may play a role in influencing the immune response. IFN-γ is an important Th1 cytokine, and the *IFN-γ* +874 polymorphism has been associated with IFN-γ mRNA and IFN-γ levels [9,20]. Moreover, the *IFN-γ* +2109A/G SNP located in intron 3 has been shown to be functional and it may eventually modify the effect exerted by the IFN-γ +874 SNP [21,22]. In this study, we investigated the influence of these two SNPs on the susceptibility of HBV-LC in a Chinese population and identified the associations between the *IFN-γ* +2109A/G polymorphisms and HBV-LC risk. The results revealed that the GG genotype and G allele of +2109A/G were associated with a significantly decreased risk of HBV-LC. Moreover, we also found that the T^+874^G^+2109^ haplotype between the +874 and +2109 locus of *IFN-γ* significantly decreased the HBV-LC risk, while A^+874^A^+2109^ haplotype significantly increased the HBV-LC risk.

With regard to HBV-LC, we found that +2109A/G GG genotype and G allele could serve as possible protective factors of HBV-LC. Some studies reported the IFN-γ polymorphisms to be associated with inflammation and fibrosis, and inflammation and fibrosis are the main factors for the development of LC [23,24]. In agreement with our study, Chevillard et al. [21] found that the +2109A/G polymorphisms were associated with periportal fibrosis in severe hepatic fibrosis patients infected by human hepatic schistosomiasis. Moreover, the +2109 locus polymorphisms could even interfere with successful therapy in HCV-infected patients [16]. We did not observed any significant association between +874 T/A polymorphisms and HBV-LC risk. On the contrary, a study by Bouzgarrou et al. [18] found that the TT and TA genotypes of +874 T/A were associated with an approximately 2.5-fold risk of progression to HCV-LC and possibly to HCC in Tunisian populations. Dai et al. [15] also found that the T allele of +874 IFN-gamma was an independent factor associated with HCV-LC. These two studies contradict our conclusion. Actually, we found that the AA genotype frequency was significantly higher in the Chinese population than in the Tunisian population (67.1% vs. 32%). By contrast, the TT genotype frequency in the Tunisian population was much higher than that in the Chinese population (22.3% vs. 5.8%). These results suggest that genetic background plays an important role in the development of HBV-LC.

With regard to CHB, IFN-γ is considered to play a functional role in viral loading [19], consistent with its known role in the inhibition and replication of HBV-infected cells. However, we did not find any significant association between the two SNPs of the IFN-γ polymorphisms and CHB susceptibility. Concordant results were reported by Arababadi et al. [12], who also found that the IFN-γ +874 polymorphisms with serum level did not affect the risk of HBV-infected patients. Moreover, other studies were also unable to find any significant association on this topic [13,25,26]. Nevertheless, Ben-Ari et al. [27] reported the genetic ability to produce low levels of IFN-γ and the susceptibility to develop chronic HBV infection. Gao et al. [28] found that the IFN-γ +874 T/A AA genotype is a possible risk to chronic HBV. A recent meta-analysis by Sun et al. [29] indicated that the + 874 T/A TT genotype and the T allele reduced the risk of CHB among Asians. The findings about the associations between IFN-γ polymorphisms and CHB risk are still conflicting.

A haplotype is a set of closely linked genetic markers present in one chromosome that tends to be inherited together more frequently than expected by chance. With respect to IFN-γ SNPs, rs2430561 was found to be in strong LD with rs2069727 (r^2^ = 0.9, r^2^ > 0.8 cutoff defined by the HapMap Project) and CA(16) repeat in intron 1 (r^2^ = 1.0) [9,30], and rs1861494 absolute LD with rs1861493 (r^2^ = 1.0) [31]. Our results indicated that rs2430561 and rs1861494 are not within a region of strong LD (r^2^ = 0.000), which were similar with the Brazil populations (r^2^ < 0.2) [30], although these two sites located in the IFN-γ gene are adjacent to each other. Therefore, the haplotype analysis was further performed and we found T^+874^G^+2109^ was a protective haplotype and that it significantly decreased the HBV-LC risk, while A^+874^A^+2109^ haplotype significantly increased the HBV-LC risk, which further supported our conclusions and could better explain the observed associations compared with each polymorphism independently. However, note that the T^+874^G^+2109^ haplotype frequencies were less than 2% (0.7%), and the statistical power was relatively limited.

Genetic background may be distinct among different populations. The genotype frequencies of the +2109 locus in Guangxi populations were consistent with those of the HCB population. However, the AA genotype frequencies of +874 and +2109 locus were higher than those of other races. Our previous studies also found the genotype and allele frequencies of cytokines gene in Guangxi populations were quite different from other regions and ethnicities [4-7]. These results not only could partly explain the inconsistent results in different regions and races but are also the reason why we conducted this study in Southern Guangxi, China.

Our study has some limitations. First, HBV-LC is a disease need sufficient age for the development, therefore, it is understandable that the age of HBV-LC patients in the current study is higher than HBV carriers and CHB patients. Although binary logistic regression analyses adjusted for age was used, we should notice the potential affect when interpret the results in this study due to the selection bias in age. Second, because of the lack of clinical characteristics of the patients, the heritability of this trait was not discussed. Third, the current study included a relatively small sample size and statistical power. Therefore, a larger study, especially a genome-wide association study, should be conducted in the future to confirm our results. At last, the study population was limited to the Guangxi population, and thus the findings could not be generalized to other populations.

## Conclusions

To the best of our knowledge, this study is the first to determine the association between the *IFN-γ* +874 T/A and +2109A/G polymorphisms in HBV-LC risk in China. Our results found that GG genotype and G allele of +2109A/G were associated with a significantly decreased risk of HBV-LC but not for CHB in the Chinese population, and the T^+874^G^+2109^ of the two SNPs is a protective haplotype while the A^+874^A^+2109^ haplotype increased the HBV-LC risk. Further studies with a larger sample size are required to confirm the results in different regions and races.
